# Study of suspension grafting process of polypropylene

**DOI:** 10.1080/15685551.2018.1503147

**Published:** 2018-09-16

**Authors:** Fengchun Liang, Hao Yuan, Qing Shao, Wenbo Song

**Affiliations:** Division of New Products Development, Sinopec Beijing Research Institute of Chemical Industry, Beijing, China

**Keywords:** Suspension grafting, polypropylene, grafting efficiency, initiator

## Abstract

Grafting efficiency is an important indicator of polypropylene grafting reaction. A series of studies have been accomplished, including the conditions of free radical presence on PP backbone during peroxide initiation, the effect of preheat treatment and reaction time on PP suspension grafting results, and the effect of peroxide residue on properties of modified PP. An optimized grafting process was proposed by mixing and preheating polypropylene (PP) and the initiator benzoyl peroxide (BPO) before adding the grafting monomer glycidyl methacrylate (GMA),resulting in an increase in grafting efficiency from 50.0% to 78.2%. With initiator residue removed by alternating temperature treatment, the suspension grafting reaction time could be substantially reduced.

## 1. Introduction

Polypropylene (PP) is one of the most widely used plastics due to its excellent chemical and physical properties[1–4]. However, since PP is a nonpolar polymer, its colorability, adhesiveness and compatibility with polar materials are comparatively poor, which seriously hindered its application[5,6]. In order to broaden the application of PP, modification of polarity is needed. The modification of PP comprises physical modification[7–9] and chemical modification[10–12].

Among chemical modifications, grafting modification is the most effective method to overcome the drawbacks mentioned above. In grafting modification, polar monomers are chemically linked to PP backbone, resulting a greatly enhanced bonding durability between PP backbone and grafted side chains. Up to now, many methods have been developed to prepare PP-based grafting polymers, established approaches include melt grafting[13–16], solution grafting[17,18], solid phase grafting[19–23] and suspension grafting[24–27]. Melt grafting and solution grafting require high reaction temperature over 100 °C. The degradation of PP via β hydrogen elimination occurs at such a high temperature[28,29]. In addition, organic solvents used in these reactions pose a threat to the environment. Suspension grafting is usually performed in water. It is an efficient and environmental friendly grafting method with the advantages such as nonuse of organic solvent, low reaction temperature, simple equipment and technology, low degradation degree of PP, controllable reaction conditions and easy postprocessing. Therefore, suspension grafting is worth attention and researches.

Improving grafting percentage and grafting efficiency, which are two important indicators of grafting polymerization to researchers. In this paper, the influence of preheating on the grafting effectiveness in the process of BPO initiated PP suspension grafting was systematically studied. Compared with no preheating process, the grafting percentage and grafting efficiency are effectively improved, and the reaction time is reduced.

## 2. Experiment

### 2.1 Materials

PP particles (T30S) were supplied by Sinopec. The Melt Flow Rate (MFR) is 3.7 g/10 min measured at 2.16 kg at 230°C. The initiator BPO was purchased from Xi Long Chemical. GMA and methyl methacrylate (MMA) were purchased from Beijing J&K Technology Co (Beijing, China). Acetone was purchased from Sinopharm Chemical Reagent Co (Beijing, China). Ethyl acetate was purchased from Beijing J&K Technology Co (Beijing, China). GMA was distilled under reduced pressure before used to remove hydroquinone inhibitor. Other starting materials were used without further purification unless otherwise noticed.

### 2.2 Instruments and measurements

The Fourier transform infrared (FT-IR) spectra of PP-g-GMA were recorded with a Nicolet IR200 FT-IR spectrometer. Melt mass flow rate measurement (MFR) was measured by a CEAST7026 melt indexer. Measurement was performed at 230 °C with a load of 2.16 Kg. To reduce the degradation of PP at high temperature, about 0.12 g antioxidants were mixed with 10 g PP. Then the mixtures were stirred for 10 seconds. Electron paramagnetic resonance (EPR) spectra was recorded on a JES-FA200 spectrometer. The molecular weights of PP and grafted-PP were measured at 150°C by a Waters GPC 2000 instrument with o-dichlorobenzene as the solvent.The grafting percentage (GP) and grafting efficiency (GE) were calculated as per the following equations:

(1) $$GP=\frac{w_{1}-w_{0}}{w_{1}}\;\;\times \;100\%$$

(2) $$GE=\frac{w_{1}-w_{0}}{w_{2}}\;\times 100\%$$

Where $w_{0\;}$is the mass of the pristine PP particles, $w_{1}$ is the mass of grafted PP particles after extraction, $w_{2}$is the mass of monomer.

### 2.3 Grafting process

The aqueous-phase suspension grafting polymerization was carried out in a 1 L four-necked round-bottom flask equipped with a twin-blade mechanical stirrer operated at 200 rpm, a condenser tube, a nitrogen purging inlet and a thermometer. PP granules was added to the flask, and stirred for a period of time with gentle nitrogen flow to eliminate oxygen; Certain amount of BPO was dissolved in acetone, and then the solution was added into PP spheres evenly, the mixture (PP and BPO) was stirred in nitrogen atmosphere; Then, the reactor was heated up to 48 °C to remove acetone with nitrogen flow; PP particles were stirred for a period of time in nitrogen atmosphere. Then, certain amount of grafting monomer was added evenly into the flask, about 200 mL DI water (ultrasonic deoxygenation, 90 °C) was added to the flask. The grafting polymerization proceeded at 90 °C for a certain period of time. After polymerization the primary grafted product was obtained by the filtering and drying process. Then, the grafted PP spheres were extracted with ethyl acetate in a Soxhlet extractor for 24 h and dried to constant weight in vacuum oven at 50 °C, pure product was obtained.

## 3. Results and discussion

### 3.1 Influence of preheating on grafting

PP suspension grafting polymerization experiments have an usual route as follow: initiator, grafting monomers and PP are mixed at a certain temperature, then heated to the desired reaction temperature for reaction[30,31]. However, an obvious drawback in this reaction route is that the initiator will partially dissolve in grafting monomers and result in serious monomer self-polymerization side reaction and reduce grafting efficiency. In addition, the diffusion-rate of monomer is strongly dependent on the temperature and increase with higher temperatures[32–35], if monomers and PP are mixed at a low temperature, the slow diffusion of the monomers also tends to produce self-polymers, which reduces the grafting efficiency. Herein, this process has been optimized in this study to improve GP and GE.

**Scheme 1.**
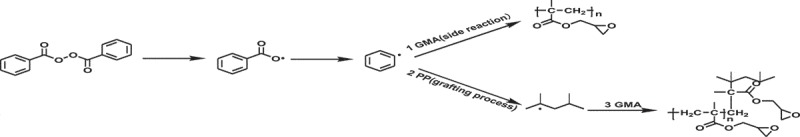
The graft process of polypropylene initiated by BPO.

The mechanism of polypropylene grafting reaction has been studied by many scholars and it has been well established that a free-radical grafting process starts with the formation of macroradicals along the PP chains[15,36–38]. Generally, the primary radicals are formed by decomposition of the initiator in the free-radical grafting system. Then the primary radicals generate a macromolecular radical by hydrogen abstraction from the backbone of PP. The macromolecular radicals could react with the monomers and form a target molecule[31]. Based on these studies, the process of PP suspension grafting initiated by BPO can be summarized as follows:

It can be seen from the grafting mechanism that the initiator is first broken down into primary free radicals, then the primary free radicals capture hydrogen on the polypropylene macromolecule chains to generate polypropylene macromolecular radicals, finally the macromolecular radicals react with the grafting monomers to generate polymerization products. Some of these free radicals also initiate homopolymerization of monomers. Therefore, when initiator, grafting monomers and PP are mixed together in the first time, the initiator inevitably dissolve in the grafting monomers and rapidly initiate self-polymerization of the grafting monomers (reaction 1 in Scheme 1), GP and GE will be significantly reduced. In this study, this process has been optimized. To be specific, PP and BPO were first added to the flask for preheating. During this process, PP macromolecular radicals were produced (reaction 2 in Scheme 1).Then the grafting monomer GMA was added in to react with macromolecular radicals (reaction 3 in Scheme 1). This reaction route not only reduced the dissolution of the initiator in monomer, but also accelerates the diffusion-rate of monomers, which maximally prevented the occurrence of monomer self-polymerization side reaction (reaction 1 in Scheme 1), and improved grafting efficiency.

**Figure 1.**
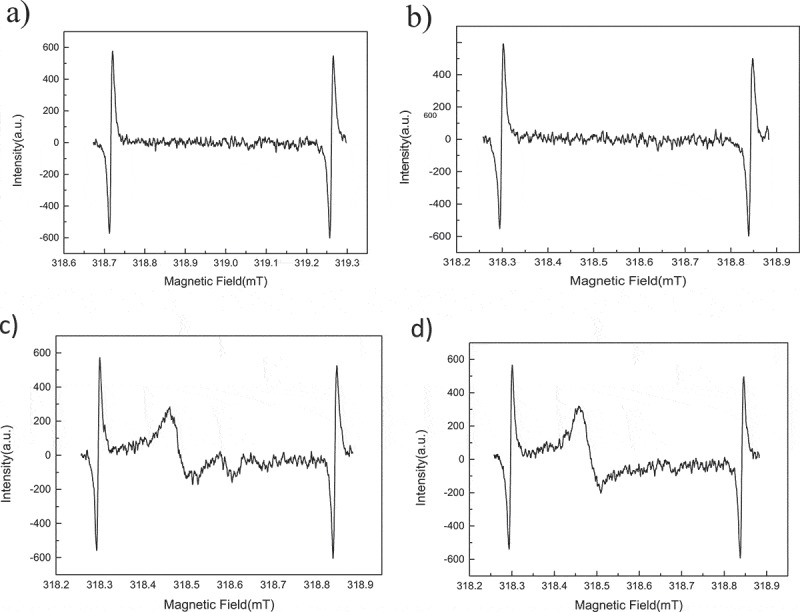
EPR spectrum of a) pure BPO; b) pure PP; c) PP initiated by BPO after 4 minutes; d) PP initiated by BPO after 54 minutes.

In order to verify whether PP and BPO can generate macromolecular radicals during preheating process, EPR measurement (Figure 1) was implemented to detect the existence of free radicals[39–41]. EPR tests were performed on several samples in a nitrogen atmosphere at a temperature of 90 °C, and the samples are pure PP, pure BPO and their mixture. The detailed test conditions were summarized in Table 1. Figure. 1 shows the EPR spectra of these samples. When only the initiator BPO presented in the system, no free radicals were detected after heated to 90 °C due to the radicals from the decomposition of BPO are not stable, neither did PP generate free radical when heated to 90 °C. When BPO and PP were mixed together, a clear signal of free radicals was detected after heated to 90 °C.

**Table 1.** Samples and test conditions for EPR.

Sample	Temperature(°C)	Atmosphere
BPO	90	N_2_
PP	90	N_2_
BPO+ PP(mBPO:mPP = 0.125:100)	90	N_2_

EPR test showed that PP macromolecular radicals can be generated by preheating. Basing on the result, we designed some experiments to verify whether the preheating process could improve the grafting efficiency. We employed GMA and MMA monomers to graft onto PP with both preheating and non-preheating methods. The results of both experiments are listed in Table 2, it is obvious that PP suspension grafting efficiency could be improved through the preheating process.

**Table 2.** Effect of preheating on grafting efficiency.

Monomer	Method	GE (%)
GMA	non-preheating	50.0
	preheating	78.2
MMA	non-preheating	78.0
	preheating	85.0

**Figure 2.**
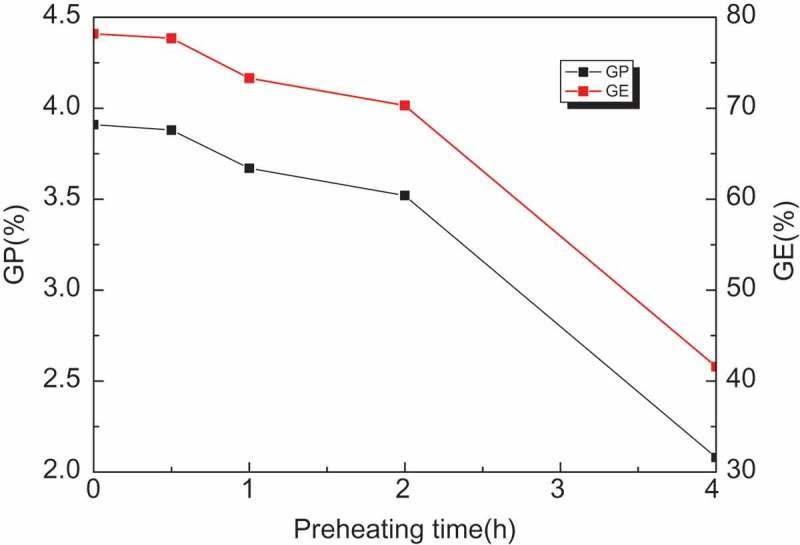
Effect of Preheating time on GP and GE.

Since preheating process is an effective method to improve grafting efficiency, we further investigated the effect of preheating time on grafting effect of PP grafted GMA. As shown in Figure 2, along with the increasing preheating time, GP and GE remained unchanged in the first half hour. Within 2 hours, grafting efficiency and grafting rate decreased slightly. After two hours, grafting efficiency and grafting percentage dropped significantly. This shows that the active sites on polypropylene were stable for a relatively long time under non-oxidation conditions. As time increase, the sites will be inactivated with the free radical generated by the initiator or polypropylene. Preheating experiments have shown that when the preheating time did not exceed 0.5 hours, the optimal grafting percentage and grafting efficiency can be achieved.

Above research on preheating process fully shows that it can effectively improve grafting percentage and grafting efficiency of PP suspension grafting, and the optimal grafting effect can be achieved with a preheating time less than 0.5 hours.

### 3.2 Influence of reaction time on grafting

**Figure 3.**
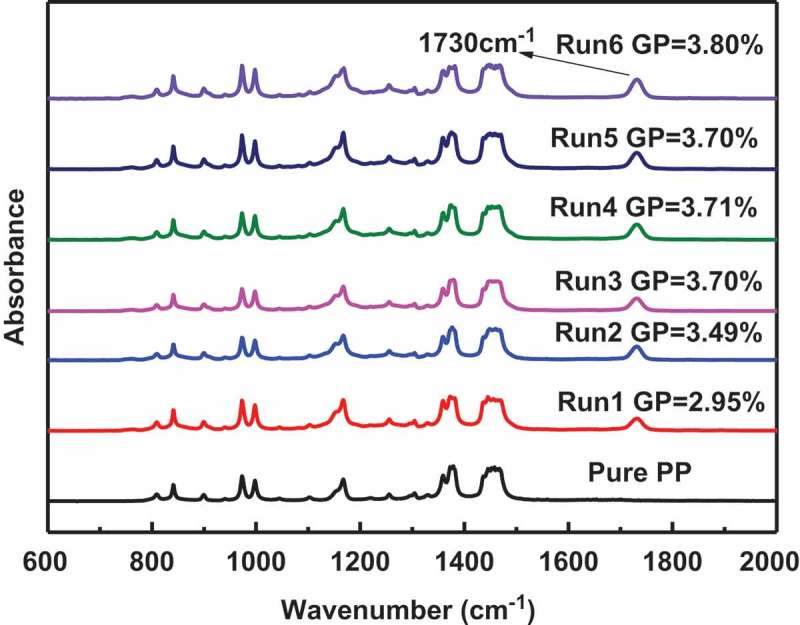
FT-IR spectra of pristine PP and PP-g-GMA with different reaction time.

In general, the total reaction time for grafting polymerization is about at least four times the half-life of the initiator at the reaction temperature[42,43]. Since the half-life of BPO at 90 °C is 1 h, required reaction time for BPO grafting reaction should be 4 h. However, for a chemical process, it is helpful to reduce the reaction time to reduce cost and maximize profit. Thus, we investigated the effect of reaction time on PP grafted GMA to optimize the reaction process, taking GP and GE as standards to measure the quality of the grafting reaction. Table 3 shows the results of the PP-g-GMA experiments with different reaction times. It can be seen that GP and GE were almost constant when the reaction time exceeded 1 h, this indicates that the reaction was complete in the first hour. To further verify this conclusion, FT-IR measurement was used on (Figure 3) the PP-g-GMA samples (after extracted with ethyl acetate) with different reaction times. Compared to the spectrum of pristine PP, strong absorption bands appeared at 1730 cm^−1^ in the grafting products with different reaction times. This characteristic band was attributed to the carbonyl group stretching of GMA. Since the monomer and homopolymer of GMA had been removed by extraction, it can be guaranteed that GMA monomer was successfully grafted onto PP backbone. It can be seen that when the reaction time exceeded 1 hour, the area of the characteristic peaks of the grafted samples is almost the same, which indicates that optimal grafting percentage can be achieved after 1 hour. This is consistent with the previous conclusion that the optimal grafting percentage and grafting efficiency can be achieved after one hour of reaction.

**Table 3.** GP and GE of different reaction time.

RUN	PP (g)	GMA (g)	BPO (g)	H_2_O (g)	Reaction time (h)	GP (%)	GE (%)
1	100	5	0.125	200	0.25	2.95	61.0
2	100	5	0.125	200	0.5	3.49	72.4
3	100	5	0.125	200	1.0	3.70	75.4
4	100	5	0.125	200	1.5	3.71	77.0
5	100	5	0.125	200	2.0	3.70	76.2
6	100	5	0.125	200	4.0	3.80	78.2

### 3.3 Influence of peroxide residue on grafting

MFR is also an important measure of grafting effect. The MFR data of pristine and grafted PP are listed in Table 4. As shown, the MFR value of grafted PP is lower than that of pristine PP. It is mainly because grafting polymerization leads to the increase of molecular weight of PP(Mn increased from 4.9 × 10^4^ to 5.7 × 10^4^ after PP grafted GMA）, and the side chains also affect the fluidity of PP, which reduces the MFR values.

**Table 4.** MFR data of pristine and grafted PP.

		Reaction Time (h)							
Samples	PP	0.25	0.5	1.0	1.0	1.0	1.5	2.0	4.0
MFR（g/10 min）	3.7	3.4	3.1	3.3	3.1	2.8	3.2	2.7	2.6

According to the study on reaction time, GP is basically the same after reaction time exceeds 1 h. However, the MFR values of the products obtained after 1 h and 1.5 h are higher than the values of the products obtained after 2 and 4 h of reactions. It is noteworthy that the MFR values of the three products obtained after 1 h are also different. We assume that these differences are from the presence of BPO residue in the products from 1 h of reactions, which may cause degradation of PP during the MFR test. However, there is no BPO residue in the product obtained after 2 and 4 hours because they have sufficient time to inactivate the BPO residue. A series of experiments have been designed to verify this assumption. First, experiments were designed to verify whether BPO remained in the product after an hour of reaction: (1)An additional amount of antioxidant was added to the sample during MFR testing. It was found that the MFR value of the product decreased from 3.2 g/10 min to 2.9 g/10 min. The decrease of MFR value indicates that the antioxidant effectively inhibited PP degradation caused by residual BPO, confirming the presence of BPO residue in the product. (2) The product obtained after 1h of reaction was poured into water and heated for three hours. At this point, the MFR values of the product should be reduced as residual BPO has been consumed during the heating process. The test results approved this assumption, the MFR value of the sample decreased from 3.2 g/10 min to 2.5 g/10 min. This is also a sufficient evidence of BPO residue in the product. The above experiments demonstrate that there is BPO residue in the products obtained after one hour of reaction. Second, whether BPO residue actually causes degradation of PP needs to be proved. The proof method is to add a certain amount of BPO to PP and measure its MFR value.We find that the MFR value increased from 3.7 g/10 min to 4.1 g/10 min, which indicates that BPO residue could cause degradation of PP.

According to the analysis of GP、GE and MFR, it can be seen that the optimal GP and GE can be reached in an hour, but there was BPO residue in the product. In order to remove the residual BPO, after 1 h of reaction, the system temperature was raised rapidly to 120 °C and held for half an hour. The MFR value of the product obtained by this method is 2.5 g/10 min, showing that this process can effectively solve the problem of residual BPO in the product. By this method, the reaction time of suspension grafting GMA onto PP can be shortened to 1.5 h.

## 4. Conclusions

In conclusion, we put forward a novel kind of charging sequence for PP based suspension grafting polymerization. After conducting a preheating procedure before adding the grafting monomer, the grafting percentage and grafting efficiency of grafting polymerization was greatly enhanced. When GMA was used as the grafting monomer, the preheating method increased the grafting efficiency from 50.0% to 78.2%, and the optimal grafting percentage and grafting efficiency can be achieved after one hour of reaction. The heating process can effectively solve the BPO residue problem and shorten the reaction time to 1.5h. The result demonstrates an efficient way to improve reaction efficiency and reduce reaction time, which is of great significance to the future industrialization of suspension grafting polymerization.
